# Supplementary material for: Causal Relationship between Aging and Anorexia Nervosa: A White-Matter-Microstructure-Mediated Mendelian Randomization Analysis
Source: Biomedicines. 2024 Aug 16;12(8):1874. doi: 10.3390/biomedicines12081874 (PMC11351342; doi:10.3390/biomedicines12081874)
Supplement: Supplementary file 1 [file biomedicines-12-01874-s001.zip › Supplementary Figure/Figure S4/Singe SNP plot of MR analysis for TL to ubm-b-1622.pdf]

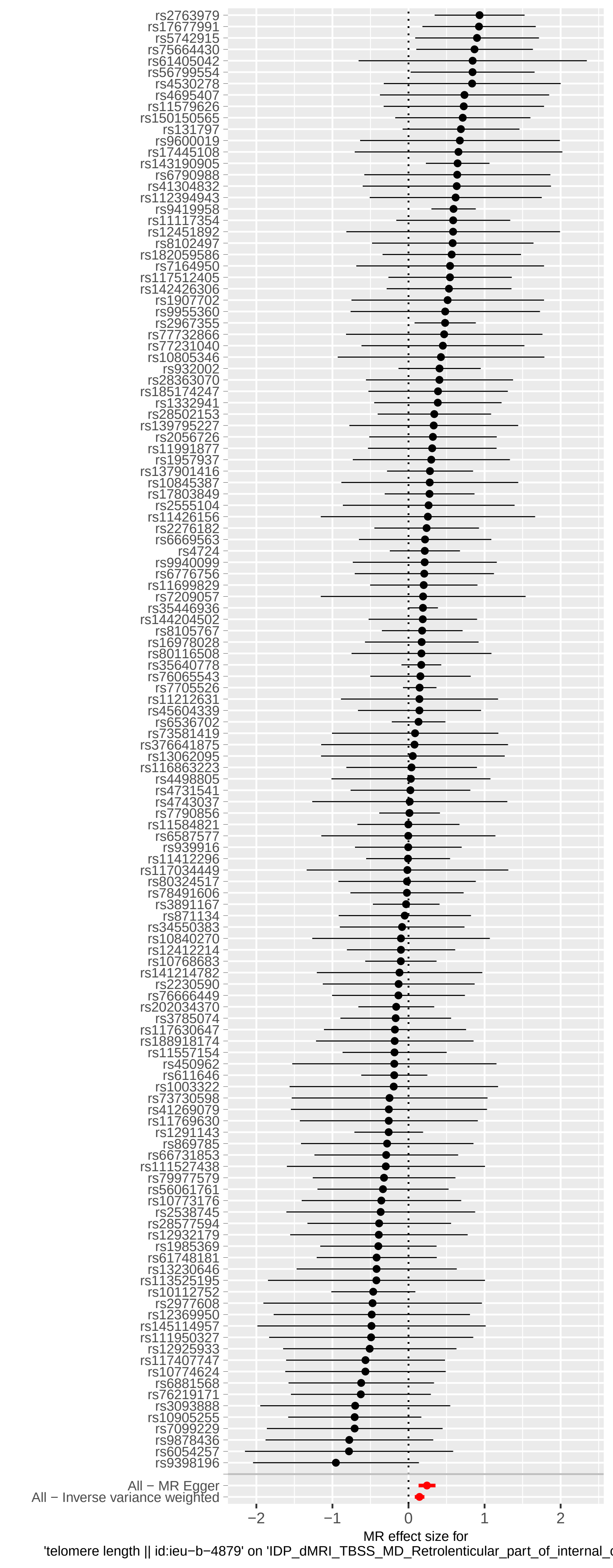
rs2763979
rs17677991
rs5742915
rs75664430
rs61405042
rs56799554
rs4530278
rs4695407
rs11579626
rs150150565
rs131797
rs9600019
rs17445108
rs143190905
rs6790988
rs41304832
rs112394943
rs9419958
rs11117354
rs12451892
rs8102497
rs182059586
rs7164950
rs117512405
rs142426306
rs1907702
rs9955360
rs2967355
rs77732866
rs77231040
rs10805346
rs932002
rs28363070
rs185174247
rs1332941
rs28502153
rs139795227
rs2056726
rs11991877
rs1957937
rs137901416
rs10845387
rs17803849
rs2555104
rs11426156
rs2276182
rs6669563
rs4724
rs9940099
rs6776756
rs11699829
rs7209057
rs35446936
rs144204502
rs8105767
rs16978028
rs80116508
rs35640778
rs76065543
rs7705526
rs11212631
rs45604339
rs6536702
rs73581419
rs376641875
rs13062095
rs116863223
rs4498805
rs4731541
rs4743037
rs7790856
rs11584821
rs6587577
rs939916
rs11412296
rs117034449
rs80324517
rs78491606
rs3891167
rs871134
rs34550383
rs10840270
rs12412214
rs10768683
rs141214782
rs2230590
rs76666449
rs202034370
rs3785074
rs117630647
rs188918174
rs11557154
rs450962
rs611646
rs1003322
rs73730598
rs41269079
rs11769630
rs1291143
rs869785
rs66731853
rs111527438
rs79977579
rs56061761
rs10773176
rs2538745
rs28577594
rs12932179
rs1985369
rs61748181
rs13230646
rs113525195
rs10112752
rs2977608
rs12369950
rs145114957
rs111950327
rs12925933
rs117407747
rs10774624
rs6881568
rs76219171
rs3093888
rs10905255
rs7099229
rs9878436
rs6054257
rs9398196
All − MR Egger
All − Inverse variance weighted
−2
−1
0
1
2
MR effect size for
'telomere length || id:ieu−b−4879' on 'IDP_dMRI_TBSS_MD_Retrolenticular_part_of_internal_c
